# Emotion recognition through static faces and moving bodies: a comparison between typically developed adults and individuals with high level of autistic traits

**DOI:** 10.3389/fpsyg.2015.01570

**Published:** 2015-10-23

**Authors:** Rossana Actis-Grosso, Francesco Bossi, Paola Ricciardelli

**Affiliations:** ^1^Department of Psychology, University of Milano-BicoccaMilano, Italy; ^2^Milan Centre for NeuroscienceMilano, Italy

**Keywords:** emotions recognition, faces, biological motion, point-light displays, Autism Spectrum Disorders, Asperger Syndrome, Autism Spectrum Conditions

## Abstract

We investigated whether the type of stimulus (pictures of static faces vs. body motion) contributes differently to the recognition of emotions. The performance (accuracy and response times) of 25 Low Autistic Traits (LAT group) young adults (21 males) and 20 young adults (16 males) with either High Autistic Traits or with High Functioning Autism Spectrum Disorder (HAT group) was compared in the recognition of four emotions (Happiness, Anger, Fear, and Sadness) either shown in static faces or conveyed by moving body patch-light displays (PLDs). Overall, HAT individuals were as accurate as LAT ones in perceiving emotions both with faces and with PLDs. Moreover, they correctly described non-emotional actions depicted by PLDs, indicating that they perceived the motion conveyed by the PLDs *per se.* For LAT participants, happiness proved to be the easiest emotion to be recognized: in line with previous studies we found a happy face advantage for faces, which for the first time was also found for bodies (happy body advantage). Furthermore, LAT participants recognized sadness better by static faces and fear by PLDs. This advantage for motion kinematics in the recognition of fear was not present in HAT participants, suggesting that (i) emotion recognition is not generally impaired in HAT individuals, (ii) the cues exploited for emotion recognition by LAT and HAT groups are not always the same. These findings are discussed against the background of emotional processing in typically and atypically developed individuals.

## Introduction

Research on emotion recognition has been dominated by studies focusing on faces and using *static stimuli*, in particular static photographs of facial expressions. This is probably due to two reasons. First, the recognition of facial emotional expressions is efficient with both static and moving images (although facial motion increased the likelihood of the recognition of basic expressions, Bassili, 1978, 1979), whereas this is not true with other body parts, in which emotion recognition is far more efficient with dynamic stimuli (e.g., Atkinson et al., 2004). Second, since the seminal study by Ekman et al. (1969), there is well-documented evidence that through facial expressions the human face has evolved as a major signaling and communication channel for emotions. For several decades scientists seemed to have ignored the fact that emotions are expressed and communicate to others with the whole body, which without a doubt means with faces, but also with hands, body postures, velocity of gait, tone and volume of the voice, and so on (i.e., body language). In recent years, however, an increasing number of scientists have become aware of the fact that facial expressions are not the only source of input that conveys emotionally relevant information and there is a small but consistent corpus of research showing that human observers are able to distinguish at least a limited set of emotions from static body expressions in the absence of facial cues (see Atkinson, 2013 for a review).

Emotion processing and emotion recognition have been widely investigated not only in typically developed (TD) individuals, but also in pathological populations, with a particular emphasis on people with Autism Spectrum Disorders (ASDs) or Autism Spectrum Conditions (ASCs). According to DSM-V (American Psychiatric Association [APA], 2013), ASD refers to a set of complex, polygenetic neurodevelopmental disorders, which are characterized, among other symptoms, by social and communication deficits. Recently, the broader label of ASC has been used to characterize difficulties in social and communication functioning alongside repetitive behavior and restricted interests (Baron-Cohen et al., 2009; Ashwin et al., 2015) and includes ASDs. This point of view assumes that ASDs lie on a continuum of social-communication disability which in the general population goes from no impairments to pathological conditions (Baron-Cohen et al., 2001). This view does not support the idea of diagnostic categories of autism but assumes that any person may have “autistic traits” or what has been called “the broader autism phenotype” (Bailey et al., 1995). Therefore, signs of social and communication deficits can be found even in individuals who have not received a formal diagnosis of ASC but present a high level of autistic traits. Even though diagnostic criteria for autism do not require a difficulty in the identification of emotional cues, it is commonly assumed that emotion recognition difficulties are present in individuals with ASC and they may also be present in individuals with “High Autistic Traits” (considered as part of a broader continuum). As in TD individuals, the majority of works on emotion processing in ASC has focused mainly on faces and has used static stimuli.

Although during past decades there has been a growing interest toward the role of facial movement in emotional expressions, the results are controversial, given that it is very hard to separate experimentally the processing of facial identity from that of emotional expressions. In an attempt to reduce non-motion cues, researchers have typically employed point-light or patch-light displays (hereinafter referred to as PLDs) of human bodies (biological motion), in which static form information is minimal or absent but motion information (kinematics and dynamics) and motion-mediated structural information are preserved (Johansson, 1973). PLDs, in fact, are obtained only by placing single visible markers on some crucial points (i.e., joints) of the body (or of the face in the adaptation used for studying facial motion). These displays have been proven to convey to the human observer a variety of information such as for example the nature of the action (Kozlowski and Cutting, 1978; Dittrich, 1993) and the gender of the actor (Mather and Murdoch, 1994).

There are a growing number of studies using PLDs showing a link between motion and emotion, for both faces and bodies.

While there is no consensus on whether facial motion can facilitate emotion recognition (e.g., Knight and Johnston, 1997; Bould and Morris, 2008; Fiorentini and Viviani, 2011), it is now well recognized that body language (also referred to as bodily kinematics) is sufficient for the perception of emotions (Atkinson et al., 2004; Clarke et al., 2005). This may imply that people are able to perceive emotions from kinematic patterns without having to compute the detailed shape of the human form first.

Evidence is thus accumulating regarding human ability to recognize emotions not only through photographs of facial expressions, which is documented by a lot of research, but also through (a) static body postures, (b) PLDs of moving faces, (c) PLDs of moving bodies and even through (d) PLDs of moving body parts.

Hence, if on the one hand faces are universally recognized as the major signaling and communication channel for emotions (George, 2013), on the other hand a growing body of evidence shows that bodily kinematics are also crucial for emotion recognition. The aim of the present study was therefore to investigate whether emotion recognition differs depending on whether emotions are conveyed through a static face or through body motion. In particular, our scope was to focus on the recognition of emotions on the one hand by excluding the motion component from faces, and on the other hand by concealing face identity from body motion. We hypothesized that since different emotions present different features of faces and dynamic components of body language, they may play a different role in the recognition of different emotions. We think that this comparison could also help in better understanding the process of emotion recognition in tipically developed individuals and shed some new light on ASC, and for this reason we tested both TD individuals and young adults with high functioning ASCs. In fact, it is possible that the static components of faces and the dynamic components of the human body could contribute differently as cues in the recognition of different emotions, and that the role of these cues might differ in individuals with ASC.

Research on emotion recognition difficulties in ASC has reported very mixed results. Several studies found generalized deficits on various emotion reading tasks (e.g., Davies et al., 1994; Corbett et al., 2009), but also a significant number of papers reported no differences between typical and autistic participants (e.g., Baron-Cohen et al., 1997; Castelli, 2005; Jones et al., 2011). Research has also investigated the idea that individuals with autism might have difficulties in the recognition of just some of the six basic emotions (i.e., Happiness, Surprise, Fear, Sadness, Disgust, and Anger) rather than a generalized deficit, but also in this case the results are controversial, with some studies reporting evidence, for example, of a selective difficulties in recognizing surprise (e.g., Baron-Cohen et al., 1993) or fear (e.g., Ashwin et al., 2006; Humphreys et al., 2007; Wallace et al., 2008) and other studies that failed to replicate these findings (e.g., Baron-Cohen et al., 1997; Castelli, 2005; Lacroix et al., 2009).

In a recent meta-analysis, Uljarevic and Hamilton (2013) brought together data from 48 papers, testing over 980 participants with autism, using as stimuli both faces and bodies (and both static and dynamic stimuli). The results of this meta-analysis show that there is an emotion recognition difficulty in autism, with the recognition of happiness only marginally impaired and the recognition of fear slightly worse than that of happiness.

To date, only a few research groups have explored whether individuals with ASC are different from TD observers in body emotion perception from PLDs, but these results are not entirely consistent (see Kaiser and Shiffrar, 2009, for a review). In a series of works by Moore et al. (1997, 2007) ASC-individuals were shown to have a reduced ability, compared to controls, in verbally reporting the subjective states and emotions from PLDs, but no differences were found in reporting actions or objects. According to authors, this deficit in the ability to describe emotional body actions could be interpreted as a deficit either at a perceptual-level (i.e., people with autism do not perceive correctly the emotional information conveyed by PLD kinematics) or at a semantic-level (i.e., people with autism perceive adequately the emotional information, but fail to associate it with the appropriate descriptive words). To solve this ambiguity Atkinson (2009) used a forced choice paradigm to investigate the ability of ASC-individuals to recognize emotions or actions from PLDs. As in previous studies, Atkinson found impairment for ASC in emotion recognition. However, in contrast to Moore and colleagues, the ASC-group also revealed deficits in labeling the displayed actions from PLDs and, more generally, an elevated motion coherence threshold.

Interestingly, a central issue in explaining the impairment of ASC in recognizing emotions from PLDs concerns a more general impairment in the integration of local elements into a coherent whole. In this respect, the ability to recognize and label biological motion from PLDs, independent from its emotional content, is crucial but research on this issue has led to very mixed results. Specifically, some studies have reported ASC-related impairments in identifying biological motion from PLDs (e.g., Blake et al., 2003; Annaz et al., 2010) whereas other studies failed to reveal any ASC-related impairments (Murphy et al., 2009; Saygin et al., 2010). Although a possible explanation for these discrepant results may rest on differences in the severity of the ASC individuals who participated in the studies (see Blake et al., 2003), a recent study by Robertson et al. (2014) seems to indicate another possible reason for the incongruence. In comparing TD and ASC individuals in a series of coherent motion perception judgements, both TD and ASC participants showed the same basic pattern of accuracy in judging the direction of motion, with performance decreasing with reduced motion coherence and shorter viewing durations of the displays. However, these effects were enhanced in the ASC group: despite equal performance in the longer displays, performance was much worse than the TD group in the shorter displays, and in the decreasing stimulus coherence conditions.

To our knowledge, only two studies have tried to compare faces and bodies in emotion recognition in TD. In an fMRI study, Atkinson et al. (2012) showed participants 2s-long digital video clips displaying point-light facial or body movements corresponding to angry, happy and emotionally neutral movements. The results showed, among other things, that facial and body motions activate selectively the Facial Fusiform Area and the Extrastriate Body Area (the former coding for the static structure of faces and the latter for bodies), but no evidence was found for an emotional modulation in these areas.

While in their study Atkinson et al. (2012) were comparing *moving* PLD faces with *moving* PLD bodies, Alaerts et al. (2011) carried out a study in which *static* faces and *moving* bodies were compared, as in the present study. The main aim of Alaerts et al.’s (2011) study was to investigate potential gender differences in a series of tasks involving the recognition of some basic aspects (e.g., displayed actions or PLDs gender) from PLDs depicting body movements of a male and female actor. Additionally, they tested whether the ability to recognize emotions from bodily PLD kinematics was correlated to the ability to recognize emotions from facial cues consisting of static photographs showing the eye region, as assessed by the ‘Reading the Mind in the Eyes Test’ (revised version, Baron-Cohen et al., 2001). A strong correlation between emotion recognition from body PLDs and facial cues was found, indicating that the ability to recognize the emotions expressed by other individuals is generalized across *facial* and *body* emotion perception.

Yet, no study has ever investigated whether the static components of emotional faces and the dynamic components of body language are differently involved in the recognition of different emotions, which in fact are characterized by different patterns of facial features and bodily kinematics.

We hypothesized that bodily kinematics play a fundamental role in the recognition of some emotions, while facial expressions should be crucial in the recognition of some others. Indeed, while facial expressions of emotions such as happiness and anger are unequivocally recognized as such, facial expressions of other emotions are often confused: for example a fearful face could easily be confused with a surprised one (e.g., Smith and Schyns, 2009; George, 2013). We thus reasoned that bodily kinematics is used by the emotion recognition system to disambiguate between these emotions and for this reason we expect body language to be at least as important as static faces in the recognition of fear, also in light of the fact that fear is usually associated with behaviors such as shivering, which are better detectable through body language than in emotional faces.

On the other hand, the bodily kinematics associated with some emotions such as sadness could easily be confused with neutral kinematics. For example, body language often associates slow gait and some configural cues such as bows and reclined head with sadness. However, for some individuals the very same features can be the default posture and thus do not express any particular emotion, being neutral. We thus expected that for the recognition of sadness, facial expression would play a major role, given also that sadness is often associated with behaviors such as crying or moaning, which are better expressed in the face.

Furthermore, in the literature there are two well documented effects: the so-called happy face advantage and anger superiority effect. The former consists of happy faces being recognized (and remembered) more easily and readily than other emotional faces, such as sad or fearful faces (Leppänen and Hietanen, 2003; Shimamura et al., 2006). Regarding angry faces, the anger superiority effect concerns the fact that it is easier to detect angry faces than happy faces in a crowd of neutral ones: angry faces pop-out of crowds, perhaps as a result of a preattentive, parallel search (Hansen and Hansen, 1988). It is thus interesting to see whether the same advantages extend also to bodily kinematics. For example, in a study by Atkinson et al. (2004), in which emotion recognition was studied with PLDs and full-light displays in both static and dynamic conditions, and with different qualities of motion expressing the emotions (i.e., normal, exaggerated and very exaggerated), recognition success differed for individual emotions. In particular, it was found that disgust and anger conveyed by dynamic PLDs were more likely to be confused and mixed up with fear whereas the opposite was true for sadness and happiness, which were less likely to be confused. In contrast, in a work by Chouchourelou et al. (2006), among five different emotions, the greatest visual sensitivity was found for angry walkers, and Ikeda and Watanabe (2009) found that the detection of anger was more strongly linked to explicit gait detection than happiness. Furthermore, Atkinson et al. (2012) claimed that their pilot work indicated that angry and happy point-light movements tended to be more readily identifiable than certain other emotions for both facial and body expressions. Therefore, we reasoned that it could be possible to find an advantage for at least one emotion (i.e., happiness), given that the kinematics associated with happiness is special, being faster and smoother than all the others.

Based on previous research on both ASC and TD participants, we also hypothesized about differences between them in terms of the cues they are relying on (i.e., static facial cues or dynamic body cues) to recognize the different emotions. In particular, given that the recognition of happiness is only marginally impaired in ASC individuals (Uljarevic and Hamilton, 2013), we expected them to be as good as TD in recognizing happiness both with facial expressions and PLDs. Instead for fear, we expected a different recognition performance for the ASC and TD group, not only in the light of the worse recognition of fear found in the meta-analysis by Uljarevic and Hamilton (2013), but also on the basis of the several studies that suggest a dysfunction of the amygdala – which has a specific role in the processing of fear (Adolphs, 2008) – in autism, which could cause poor recognition of fear and other negative emotions (Baron-Cohen et al., 2000; Howard et al., 2000; Ashwin et al., 2006). Lastly, given that the detection of anger has been shown to be more strongly linked to explicit gait detection (Ikeda and Watanabe, 2009), a difference in global motion processing in ASC and TD participants could be translated into a different pattern for the recognition of anger.

To summarize, in the present study we wanted to explore several hypotheses. Firstly, we aimed to evaluate the different role of body language and emotional faces in the recognition of different emotions in TD individuals. Secondly, we wanted to unveil any differences between individuals with High Autistic Traits (HAT group) and TD individuals with Low Autistic Traits (LAT group) in recognizing emotions through static faces and PLDs. Specifically, in LAT individuals we expected (i) body language to be at least as effective as static face in the recognition of fear; (ii) sadness to be better recognized through facial expression; (iii) to find an advantage for happiness also when it is conveyed through PLDs (in close similarity with the happy face advantage); (iv) HAT individuals to be as good as LAT ones in recognizing happiness both with facial expressions and PLDs; (v) HAT individuals to rely on different cues for the recognition of fear and anger than LAT participants.

To this end, we performed an exploratory experiment in which we compared the performance (i.e., accuracy and response times) of two groups of participants (i.e., HAT and LAT group) in the recognition of four basic emotions (fear, anger, sadness, and happiness), conveyed either by static face images or by PLDs^1^.

To make sure that all participants could correctly perceive the motion conveyed by the PLDs *per se*, a control test referred as “action recognition test” (see Alaerts et al., 2011) was conducted using biological motion displays, in which the actor was performing neutral actions (e.g., rowing).

## Materials and Methods

### Participants

Twenty-five (21 males, 4 females, mean age = 22.3 years, *SD* = 2.9) TD individuals, with Low Autistic Traits (“LAT” group) and twenty (16 males, 4 females, mean age = 22.8 years, *SD* = 9.0) young adults with High Autistic Traits (“HAT” group) took part in the experiment. LAT participants were undergraduate students from the University of Milano-Bicocca who received course credits for their participation in the study. HAT participants were recruited from a community center, the “Spazio Nautilus Onlus”, and were diagnosed from different clinical teams as follows: 17 participants diagnosed with Asperger Syndrome (AS) and three diagnosed with Pervasive Developmental Disorder Not Otherwise Specified (PDD-NOS), according to DSM-IV-TR (American Psychiatric Association [APA], 2000) or ICD-10 (World Health Organization [WHO], 1992) criteria. Reliable IQ measures for 13 AS participants were obtained, (mean IQ: 118.92, *SD*: 23.392) through standardized tests, administrated by the same clinical teams who made the ASD diagnosis. Although it was not possible to obtain a formal IQ assessment from all of them the participants in the HAT group had an autonomous life and/or a job which requires a good cognitive and intellectual functioning but showed an impairment in social and communication skills. It is noteworthy that no relationship was found between IQ and biological motion perception in ASD (Atkinson, 2009). All 45 participants had normal or corrected-to-normal vision and were unaware of the purpose of the study.

### Ethical Statements

All participants gave a written informed consent before testing. The study was conducted in accordance with the ethical standards laid down in the 1964 Declaration of Helsinki and fulfilled the ethical standard procedure recommended by the Italian Association of Psychology (AIP). The study was specifically approved by the local Ethics Committee of Milano-Bicocca University.

### Apparatus and Materials

The experiment was carried out in a dimly illuminated room. Participants sat approximately 60 cm away from a 19-inch LCD monitor (acer^®^V196lb; Resolution: 1600 × 1200 pixels; Refresh rate: 75 Hz) interfaced with an Intel^®^ Core^TM^ i7-3517U 1.90 GHz personal computer equipped with a NVIDIA^®^ GeForce^®^ GT 620M Video Board.

Four emotions were tested, i.e., happiness, sadness, anger, and fear. Eight emotional faces (two for each emotion, one portraying a male and one a female, considered as two versions of the same emotion and coded, respectively, as version 1 and 2), taken from Radboud Faces Database (Langner et al., 2010) were used as static face stimuli whereas eight patch light displays (PLDs) were used as bodily PLD kinematic stimuli. In the latter, emotions were conveyed solely by biological motion, specifically by the kinematics of light patches placed on the joints of an actor (each emotion being expressed through two different motion sequences, coded, respectively, as version 1 and 2, Atkinson et al., 2004, 2012).

In the action recognition test (Alaerts et al., 2011), eight additional PLDs of white dots moving against a black background were also used as stimuli, showing eight different non-emotional actions (i.e., walking, riding a bike, jumping, painting, rowing, playing tennis, saluting, using a hoe, Atkinson et al., 2004, 2012).

A computerized version of Autism Quotient (AQ) questionnaire (Baron-Cohen et al., 2001b) was filled in by the participants at the end of the Experimental Session. The questionnaire consisted of 50 statements with 4 possible responses (True, Almost True, Almost False, False).

### Procedure

The participants were individually tested. The software E-Prime 2.0^®^ (Psychology Software Tools, Inc., Pittsburgh, PA, USA) was used for stimuli presentation and data recording.

The experiment was divided in four sessions: (i) static faces test; (ii) bodily PLD kinematics test; (iii) action-recognition test (Alaerts et al., 2011), and (iv) AQ questionnaire.

Instructions were provided verbally and also appeared written on the monitor at the start of each session.

In order to be sure that the participants could extract meaningful information from PLDs and to familiarize the participants with the task, before the experiment each participant was shown a short movie displaying PLD of a walking man.

The order of presentation of the sessions (i) and (ii) was counterbalanced across participants. Both static faces test and bodily PLD kinematics test consisted of 24 trials (8 stimuli × 3 repetitions), randomized for all participants. In these first two sessions participants were asked to indicate as fast as possible the displayed emotions by pressing different buttons on a keyboard. A forced choice paradigm was used, to avoid any interference caused by possible difference in the ability to associate the emotional information with the appropriate descriptive words. The four response options (happiness, sadness, anger, and fear) were indicated on the respective response buttons (Q-key, D-key, K-key, P-key), which were labeled with the emotion name.

The pressing of the response button started a blank interval of 1 s, followed by the next trial. Each trial was presented for a maximum duration of 6 s (i.e., 1 s for pictures and 3 s for PLDs, followed by, respectively, 5 s and 3 s of a black mask), after which the blank interval, and the next trial, automatically started.

In the action-recognition test participants had to watch a series of eight short movies (duration of 3 s), and were asked to verbally describe the displayed actions in the point light animations. Each series always started with the walking man already seen before the experimental session, while the other seven movies were presented after it in random order. Each movie was cyclically presented for a maximum duration of 5 min. Participants were instructed to press the spacebar when they were satisfied with their description, which was recorded by the experimenter. The press of the spacebar started the next trial.

Finally, for the AQ questionnaire (Baron-Cohen et al., 2001b), participants were asked to read each of the 50 sentences and to press, for each sentence, one out of four possible response keys (1-key, 2-key, 3-key, 4-key), which were labeled with the four response options (True, Almost True, Almost False, and False, respectively). The software E-Prime 2.0^®^ (Psychology Software Tools, Inc., Pittsburgh, PA, USA) was used for both questionnaire presentation and to automatically compute the questionnaire total score.

The whole experiment lasted approximately 30 min. Participants were free to interrupt the Experiment at any moment and to take a brief rest between different sessions.

## Results

### Preliminary Data Analysis

Three preliminary analyses were performed.

First, we checked for a possible effect of Repetition on Accuracy in the Emotion Recognition Test: a repeated-measures analysis of variance (ANOVA) with Emotion, Stimulus, Stimulus Number and Repetition showed no significant effect of Repetition [*F*(2,38) = 1.549, *p* = 0.226].

Second, an independent sample *t*-test on the number of correct responses for the action recognition test was carried out. It showed no difference in accuracy between LAT and HAT groups [*t*(43) = -0.542, *p* = 0.590], indicating that all participants could correctly perceive the action performed in the video and conveyed by the PLDs *per se*.

Third, the AQ scores were compared between the different experimental groups through an independent sample *t*-test to make sure that the two groups differed in terms of Autistic traits. It showed a significant effect [*t*(29.06) = -5.214, *p* < 0.001] between LAT participants (mean = 16.08, *SD* = 5.09) and HAT participants (mean = 27.55; *SD* = 8.721) confirming that the two groups indeed differed.

With regards to the main analyses, sessions (i) “static faces test” and (ii) “bodily PLD kinematics test” are hereinafter referred to as “emotion recognition test”. To compare the recognition performance in the emotion recognition test for LAT and HAT, accuracy (i.e., the proportion of correct responses) and response times were analyzed with two Mixed Models Analysis. The first four trials for each participant were considered to be practice trials and were discarded from the analysis. Degrees of freedom in Mixed Models Analyses were estimated through the Satterthwaite approximation method. In the next section the results for accuracy and response times to static faces and bodily PLD kinematics tests are separately discussed.

#### Accuracy

For both LAT and HAT participants overall classification accuracy averaged across stimulus type was high [89.5% (*SD* = 22.52) for LAT vs. 84.6% (*SD* = 25.42) for HAT].

A Mixed Models analysis with Emotion, Stimulus Type and Stimulus Version as independent within-subjects variables and Group as independent between-subjects variable showed a significant main effect of Emotion [*F*(3,129) = 15.309, *p* < 0.001]; the interactions Emotions × Stimulus Type [*F*(3,151.913) = 2.921, *p* < 0.05] and Emotion × Stimulus Type × Group [*F*(3,151.913) = 4.198, *p* < 0.01] were also significant. No other factors or interactions were significant.

The variance component of each random factor (reported in **Table 1**) can be estimated. If the estimated variance components are larger than zero, then each random factor captures a significant variance component. So this model captures data dependency due to the repeated-measure design (Gallucci and Leone, 2012).

**Table 1:** Variance of random coefficients (on Accuracy).

Parameter	Estimate	* SE*
Residual	0,035994	0,004463
Intercept	0,002855	0,001724
Emotion	0,002733	0,002760
Emotion × Stimulus	0,004865	0,003326
Emotion × Stimulus version	0,002088	0,003102
Stimulus × Stimulus version	0,002990	0,001779


**FIGURE 1:**
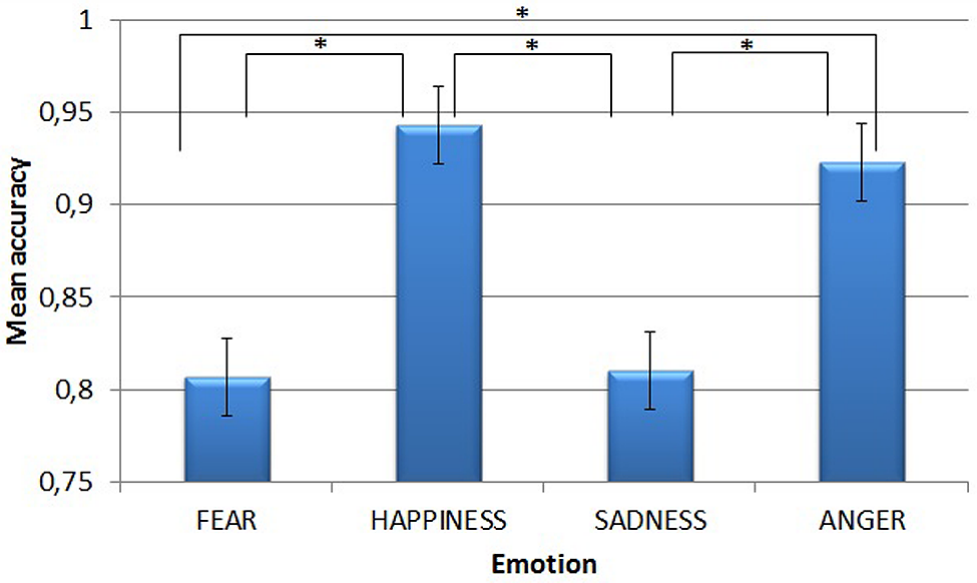
**Mean accuracy in the emotion recognition task, for each of the four emotions.** Asterisks highlight significantly different means comparisons. Error bars represent standard errors.

**Figure 1** shows the main effect of Emotion. *Post-hoc* tests (Sidak correction) revealed that accuracy for trials conveying happiness (mean = 0.943) and anger (mean = 0.923) was higher than for those conveying fear (mean = 0.807) and sadness (mean = 0.810, all *p*s < 0.001). However, both happiness and anger and both fear and sadness did not differ from each other (*p* = 0.974 and *p* > 0.999, respectively).

As stated above, the main effect of Emotion was modulated by a significant interaction with Stimulus Type, which was itself modulated by a significant 3-way interaction Emotion × Stimulus Type × Group. To follow up this significant 3-way interaction, three different Simple Effect Analyses were performed on the interaction Emotion × Stimulus Type × Group.

**FIGURE 2:**
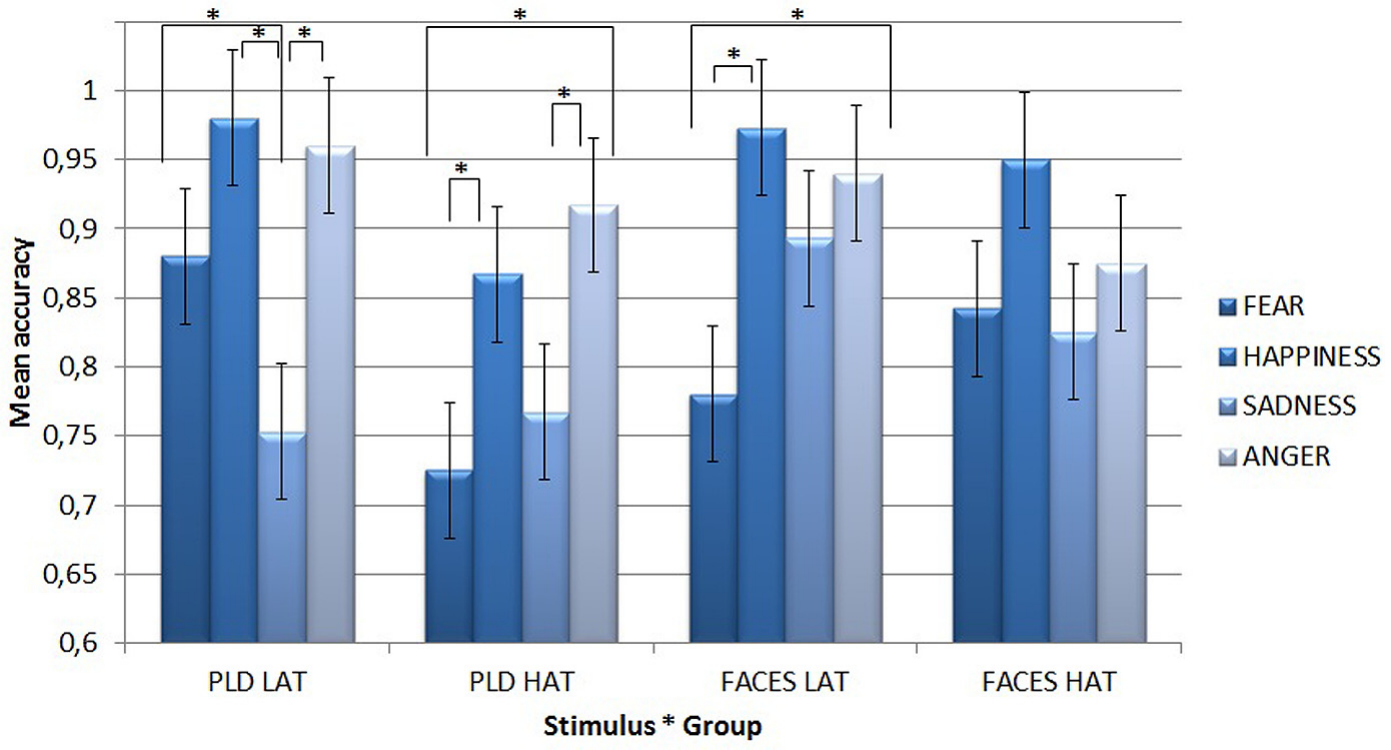
**Mean accuracy for PLDs **(left)** and static faces **(right)** in the emotion recognition task for the two groups of participants (i.e., LAT and HAT) as a function of the four different emotions.** Asterisks highlight significantly different means comparisons. Error bars represent standard errors.

A first Simple Effect Analysis compared differences among single emotions conveyed by different types of stimuli for the two groups of participants (see **Figure 2**).

With static face stimuli, LAT participants were the least accurate for fear (mean = 0.78), which differed significantly from happiness (mean = 0.973, *p* < 0.001) and anger (mean = 0.94, *p* = 0.001). By contrast, the HAT group did not show any significant difference for any emotion.

With PLDs, LAT participants were the least accurate for sadness (mean = 0.753), which differed significantly from fear (mean = 0.88, *p* = 0.038), happiness (mean = 0.98, *p* < 0.001), and anger (mean = 0.96, *p* < 0.001). HAT participants were less accurate for fear (mean = 0.725) than for happiness (mean = 0.867, *p* = 0.038) and anger (mean = 0.917, *p* = 0.001), while sadness (mean = 0.767) showed a lower accuracy in comparison with anger (*p* = 0.023).

A second Simple Effect Analysis compared the two groups’ accuracy for different emotions as a function of stimulus type. It showed that with PLDs, HAT participants recognized both fear (mean = 0.725) and happiness (mean = 0.867) less accurately (*p* = 0.004 for fear and *p* = 0.033 for happiness) than LAT participants (mean = 0.88 for fear and mean = 0.98 for happiness).

**FIGURE 3:**
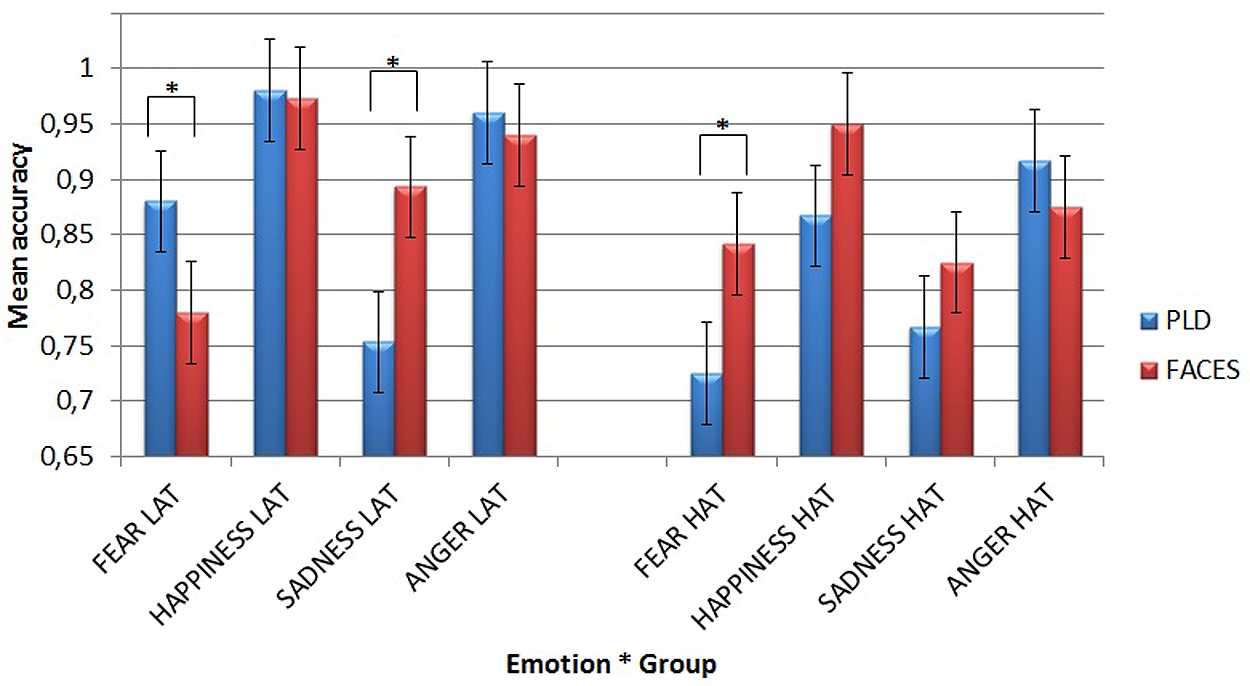
**Mean accuracy for the two different types of stimuli in the emotion recognition task as a function of different emotions and groups.** Asterisks highlight significantly different means comparisons. Error bars represent standard errors.

Finally, a third Simple Effect Analysis compared the two different kinds of stimuli as a function of different emotions in the two groups of participants (**Figure 3**).

**FIGURE 4:**
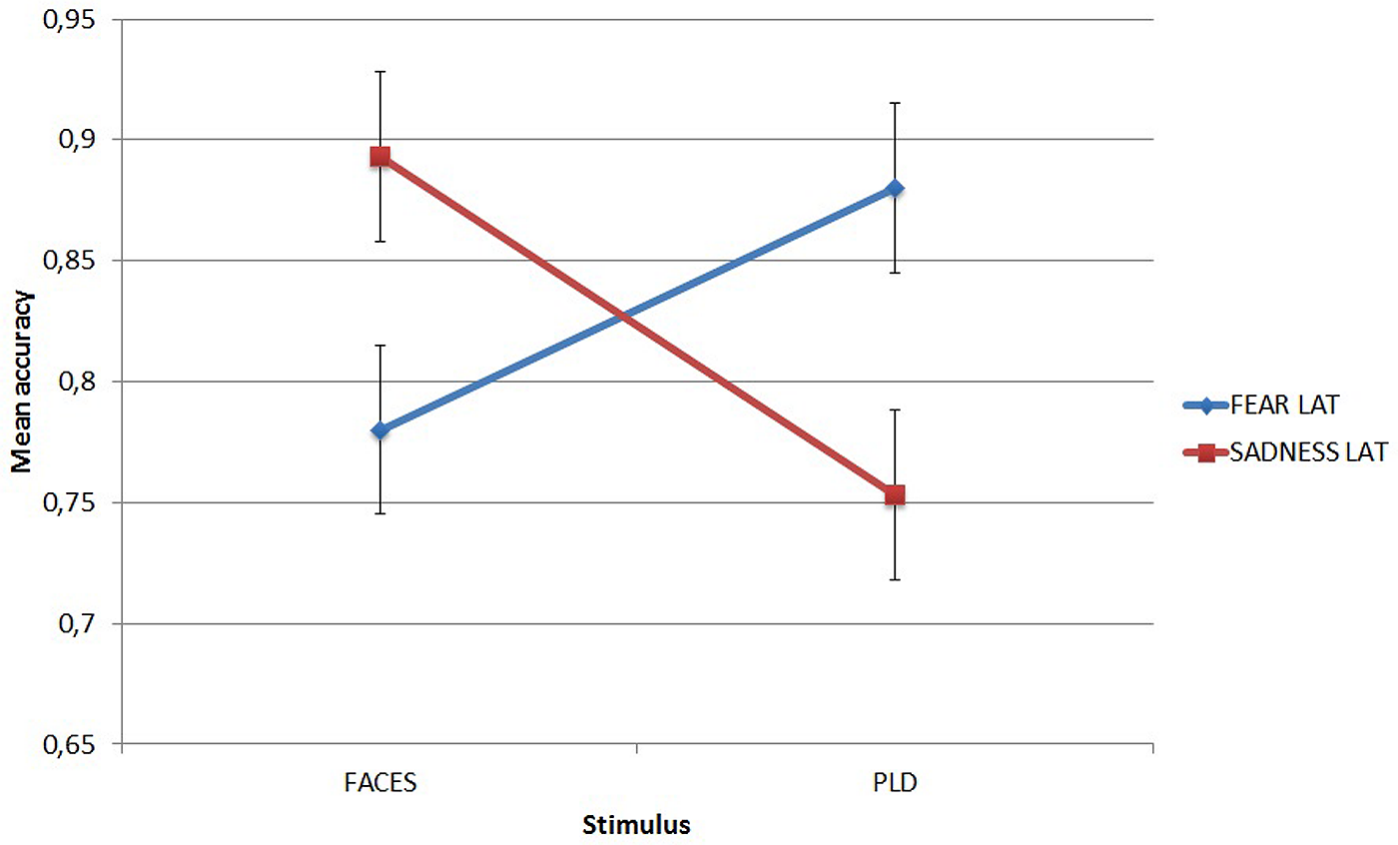
**Mean accuracy of Low Autistic Traits group in the emotion recognition task for fear and sadness as a function of stimulus type.** Error bars represent standard errors.

It showed that LAT participants recognized fear better through PLDs (mean = 0.88) than through faces (mean = 0.78, *p* = 0.025), while they recognized sadness better through faces (mean = 0.893) than through PLDs (mean = 0.753, *p* = 0.002). This dissociation can be appreciated in **Figure 4**.

By contrast, HAT participants recognized fear better through faces (mean = 0.842) than through PLDs (mean = 0.725, *p* = 0.019).

Thus, as hypothesized, while in LAT participants the recognition of fear was more accurate when it was conveyed by PLDs, HAT group showed the opposite pattern with a more accurate recognition of fear when it was conveyed by static face images.

#### Response Times

Data relative to static faces were analysed and considered separately from data relative to PLDs, given that each PLD lasted 3 s whereas each static face was presented for 1sec. Furthermore, participants were more familiar with pictures of static emotional faces than with PLDs and this implies that each PLD, at least at the first repetition, was shown for its entire duration (i.e., 3 s) whereas participants often gave their response to static faces before the entire stimulus duration.

A Mixed Models analysis with Emotion, Stimulus Type, Stimulus version and Repetition as independent within-subjects variables and Group as an independent between-subjects variable showed a main effect of both Stimulus Type [*F*(1,44.199) = 256.941, *p* < 0.001] and Repetition [*F*(2,169.615) = 16.53, *p* < 0.001], as well as a more interesting main effect of Emotion [*F*(3,144.064) = 20.294, *p* < 0.001]. The Stimulus × Repetition interaction was significant [*F*(2,169.718) = 7.787, *p* = 0.001] as well as the Repetition × Group interaction [*F*(2,169.615) = 3.237, *p* = 0.042] and the 3-way interaction Emotion × Stimulus Type × Group [*F*(3,137.029) = 2.749, *p* = 0.045]. No other interaction was significant.

The variance component of each random factor (reported in **Table 2**) can be estimated. If the estimated variance components are larger than zero, then each random factor captures a significant variance component. So this model captures data dependency due to the repeated-measure design (Gallucci and Leone, 2012).

**Table 2:** Variance of random coefficients (on RTs).

Parameter	Estimate	* SE*
Residual	301391,179437	14328,687854
Intercept	84830,759753	36639,482221
Emotion	7334,111899	11135,592106
Stimulus	103942,181184	29218,666425
Emotion ×Stimulus	21028,862804	11889,812151
Emotion ×Stimulus version	22844,915617	10577,498793
Stimulus × Stimulus version	125,272384	6603,288149
Stimulus × Repetition	17719,750719	7978,904878


The main effect of Stimulus was due to the fact that, as explained above, RTs for pictures of static faces were consistently faster than RTs for PLDs. The main effect of Repetition was modulated by significant interactions with both Stimulus Type and Group. A Simple Effect Analysis on the first interaction (i.e., Repetition × Stimulus Type) showed that RTs for PLDs at the first presentation were significantly longer (mean = 2804.077 ms) than RTs for PLDs at the second (mean = 2477.570 ms, *p* < 0.001) and the third repetitions (mean = 2402.502 ms, *p* < 0.001), which did not significantly differ from each other. On the contrary, RTs for faces did not significantly differ among the three repetitions (all *p*s > 0.5). A Simple Effect Analysis on the second interaction (i.e., Repetition × Group) showed that, while LAT participants presented a linear trend in RTs across repetitions, this was not the case for HAT participants: for LAT the first presentation (mean = 2072.365 ms) showed higher RTs than both the second (mean = 1935.685 ms, *p* = 0.059) and the third repetition (mean = 1794.718 ms, *p* < 0.001), which were also significantly different from each other (*p* = 0.014); for HAT participants only the first presentation (mean = 2088.473 ms) presented higher RTs than the second (mean = 1840.949 ms, *p* < 0.001) and the third ones (mean = 1890.212 ms, *p* = 0.002), which did not differ from each other (*p* > 0.999).

*Post hoc* tests (Sidak correction) on the main effect of Emotion showed that LAT participants’ RTs for trials conveying happiness (mean = 1676,756 ms) were lower than those conveying all the other emotions: fear (mean = 2106,651 ms, *p* < 0.001), sadness (mean = 2088,837 ms, *p* < 0.001), and anger (mean = 1864,781 ms, *p* = 0.06). Moreover, anger presented significantly lower RTs than fear (*p* = 0.003) and sadness (*p* = 0.014). For the HAT group, only happiness (mean = 1724,201 ms) showed lower RTs than all other emotions (all *p*s < 0.05): fear (mean = 2084,563 ms), sadness (mean = 1987,800 ms), and anger (mean = 1962,947 ms).

**FIGURE 5:**
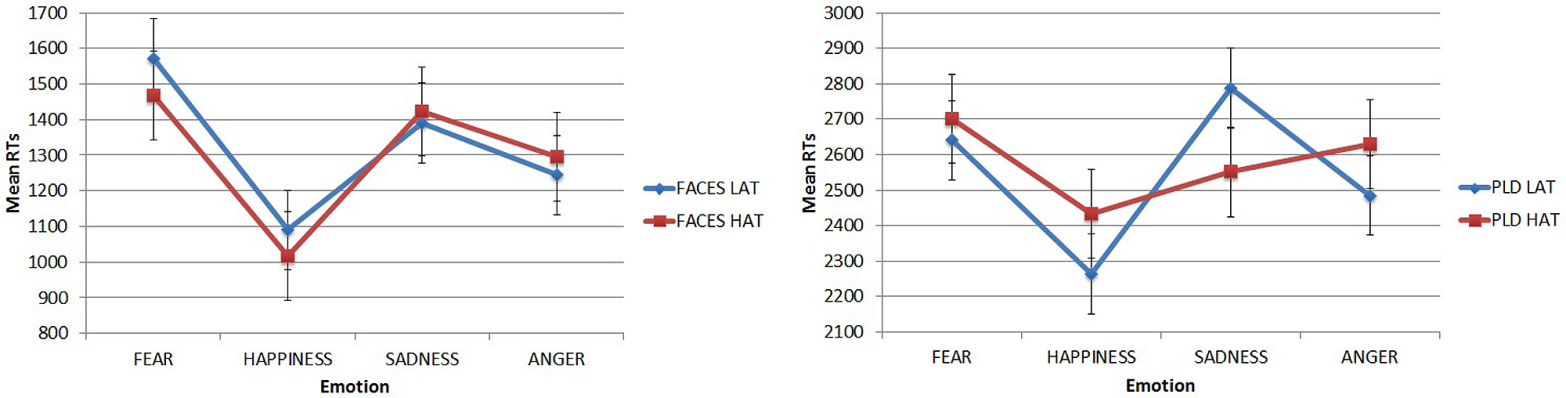
**Mean RTs in the emotion recognition task for faces **(left)** and PLDs **(right)** in the two groups of participants as a function of different emotions.** Error bars represent standard errors.

The main effect of Emotion was modulated by the 3-way interaction Emotion × Stimulus Type × Group. A Simple Effect Analysis was conducted on this 3-way interaction. For faces (**Figure 5**, left) both LAT and HAT participants showed lower RTs for happiness (mean = 1090.173 ms and 1016.228 ms, respectively, for LAT and HAT groups) than for all the others emotions: fear (LAT mean = 1572.327 ms, *p* < 0.001; HAT mean = 1468.859, *p* < 0.001) sadness (LAT mean = 1390.105 ms, *p* = 0.01; HAT mean = 1423.866 ms, *p* = 0.001) and anger (but only for HAT participants, mean = 1296,003 ms, *p* = 0.042). For LAT participants RTs for angry faces were significantly lower than RTs for fearful ones (*p* = 0.007).

For PLDs (**Figure 5**, right), LAT participants showed significantly lower RTs for PLDs conveying happiness (mean = 2263.338) than those conveying fear (mean = 2640.976, *p* = 0.001) and sadness (mean = 2787.568, *p* < 0.001); RTs for angry PLDs were also lower than those conveying sadness (*p* = 0.018). HAT participants did not show any significant advantage for PLDs.

Regarding this last comparison, it should be noted that, in principle, it is possible that the difference between RTs for different emotions with PLDs was not due to a difference in emotion recognition, but to a difference in actor performance. In other words, it is possible that PLDs conveying happiness were detected faster not because happiness is the easiest emotion to detect, but because the actor was more effective in performing that specific emotion than all the others. However, this is also true for static pictures. Nevertheless, given that studies using PLDs as stimuli for emotion recognition are not as common as studies using pictures of static faces, any comparison across different emotions with PLDs should be taken with caution and no strong conclusion should be drawn from it.

## Discussion

We investigated whether the type of stimulus (i.e., pictures of static faces vs. body motion) contributes differently to the recognition of four different emotions (i.e., Happiness, Anger, Fear, and Sadness). To this end, we performed an exploratory study aimed at comparing LAT and HAT individuals to test if the two groups based their recognition on different cues (static facial cues vs. bodily kinematics). Specifically, we were interested in seeing in LAT individuals (i) whether body language was at least as effective as static face in the recognition of fear; (ii) sadness was better recognized through facial expression; (iii) the presence of an advantage for happiness also when conveyed through PLDs. Moroever, we expected (iv) HAT individuals to be as good as LAT ones in recognizing happiness both with facial expressions and PLDs; and (v) HAT individuals to rely on different cues for the recognition of fear and anger than LAT ones.

Interestingly, the action recognition test showed no difference between the LAT and HAT group, indicating that HAT participants could correctly perceive the motion conveyed by the PLDs *per se*. This result confirms the results by both Moore et al. (1997) and Hubert et al. (2009), who reported that participants with autism were perfectly capable of integrating the individual points of the PLD into a whole, and with several other studies showing that global processing of hierarchical stimuli (i.e., the integration of local elements into a coherent whole) is not specifically impaired in people with autism (e.g., Mottron et al., 2003; Dakin and Frith, 2005, for a review). However, there are also several studies reporting ASC impairments in identifying biological motion from PLDs (e.g., Blake et al., 2003; Atkinson, 2009; Annaz et al., 2010; Nackaerts et al., 2012), and a more general deficit in ASC in coherent motion processing (Spencer et al., 2000; Milne et al., 2002). The latter deficit is typically considered as a good example of atypical global perceptual processing in individuals with ASC, given also its correlation with other markers of atypical global perception (Pellicano et al., 2005). The fact that our HAT participants could correctly perceive biological motion from PLDs is thus in disagreement with the above studies. We think that the ability of HAT participants to perceive biological motion, found in the present study, could be due either to the fact that participants in our study were all high functioning (see Blake et al., 2003) or to the fact that the time of presentation of the displays both in the action recognition test and in the emotion recognition test all lasted 3 s. Robertson et al. (2014) found that when presented with PLDs, individuals with ASC showed comparable performance to control participants only if PLDs duration was “long” (1.5 s). In contrast, impairment was found for a shorter duration (0.2 s.). It is thus possible that HAT participants exhibited similar behavioral results as LAT participants, only because the stimulus duration of the PLDs used in our study was “long enough”, while a shortening of viewing duration would cause a worsening in the performance. Another possibility is the combination of different factors. That is, our participants were high functioning without a severe deficit in global perceptual processing (or they have been rehabilitated) and the duration of PLDs was long enough to efficiently integrate the local elements into a global configuration.

The possible causes that could explain HAT individuals’ ability to recognize biological motion can also account for the fact that HAT participants were very accurate in perceiving emotions both with faces and with PLDs, being as accurate as LAT participants. The fact that our HAT participants could compensate for their possible deficits in emotion recognition makes it even more striking the fact that they relied on different cues from those used by LAT group in emotion recognition.

Overall, our results confirm that emotion recognition is not globally compromised in HAT participants – at least for our group of participants and with the type of stimuli used in the present study – since some impairment was found only for specific emotions.

However, differently from LAT participants, HAT ones did not show any significant advantage for any emotion. Happiness, in fact, proved to be the easiest emotion to be recognized only for LAT participants but not for HAT participants. In line with previous studies, for the LAT participants we found a *happy face advantage* (Leppänen and Hietanen, 2003; Shimamura et al., 2006, although no difference was found in accuracy for happiness and anger), which for the first time was also found for bodies. We propose to call this latter effect *happy body advantage*, to underline its similarity with the analogous happy face advantage (i.e., better accuracy and faster response times). One of our initial hypotheses was to find an advantage for the recognition of happiness in PLDs. Our reasoning was mainly based on the peculiarity of the kinematics associated with happiness (faster and smoother as compared to the kinematics associated to all the other emotions), and in line with previous studies (Atkinson et al., 2004, 2012). Results for LAT participants, thus, confirm our reasoning. By contrast, the results for HAT participants partially contradict one of our initial hypotheses according to which we expected HAT participants to be as good as LAT ones in recognizing happiness both with facial expressions and PLDs. In fact, HAT participants did not show the same advantage as shown by LAT ones for the recognition of happiness. Interestingly, Uljarevic and Hamilton (2013) showed a negligible impairment in the recognition of happiness in autism. This very mild impairment thus could be the reason why no advantage was shown for this particular emotion in our HAT sample.

However, for both happy face and happy body advantages in LAT participants no difference was found in accuracy between both stimulus types conveying happiness and anger. Thus, even if both happy faces and happy PLDs were recognized faster than all the other emotions (and more accurately than fear and sadness), they were not recognized more accurately than angry faces and PLDs. We think that, at least for faces, this could be due to the so-called anger superiority effect (Hansen and Hansen, 1988), for which it is easier to detect angry faces than happy faces. This effect is usually observed in visual search paradigm (in which angry faces pop out from a crowd of neutral ones), which is a typical attentive task. We speculate that, even if in a typical perceptual task as in the one tested in our study the anger superiority effect does not emerge, nonetheless angry stimuli are more perceptually (and behaviourally) salient than the ones conveying fear and sadness. For this reason they do not differentiate from the happy ones, which are easier to detect (because of the happy face advantage). Even if an anger superiority effect has never been reported for bodies, the same reasoning holds for PLDs, given that evidence of a greater visual sensitivity for angry walkers than for the other five different emotions has been reported (Chouchourelou et al., 2006). Moreover, there is a stronger link of anger, as compared to happiness, with the detection of gait in PLDs (Ikeda and Watanabe, 2009). It is thus possible that, for PLDs as well as for faces, anger is at least as perceptually salient as happiness and this would explain why a difference in accuracy between happy and angry PLDs has not been found.

For the remaining two emotions tested in this study, fear and sadness, an interesting result emerged, pointing out that certain emotions are expressed better through dynamic information than through static ones. In particular, LAT participants relied more on static faces to recognize sadness, but on PLDs to recognize fear. This is in line with our initial hypotheses. In fact we hypothesized that (i) for the recognition of sadness, facial expression would play a major role, given that the body language associated with sadness (e.g., slow gait, bows and reclined head) could be neutral (i.e., non-emotional) for some individuals (and also given that sadness is often associated with behaviors such as crying or moaning, which are better expressed with the face); (ii) for the recognition of fear, bodily kinematics would be at least as important as static faces, given that it would be used by the emotion recognition system to disambiguate between fear and surprise (which could be easily confused, see Smith and Schyns, 2009). This is also consistent with the idea that fear is usually associated with behaviors such as for example shivering, which are better detectable in body language than in emotional faces.

The advantage for motion kinematics in the recognition of fear is not present in HAT participants. In fact, for what concerns fear processing and recognition, our results show that HAT participants are often inclined to use strategies based on processing face details, which are different from those used by control participants.

Different speculations are possible to explain this result. On the one hand, one explanation could be based on the fact that adults with HAT are usually trained to recognize different emotions through faces. For this reason they could learn to compensate for a general deficit in emotion recognition, but in doing so they learn to rely more on static face details than on bodily kinematics, for which they do not undergo any specific training. This would explain why, when LAT individuals use kinematic cues to recognize fear, our HAT individuals do not rely on these cues.

Another possible explanation refers again to a lack of confidence in bodily kinematic cues for HAT individuals, but does not refer neither to a deficit in emotion recognition nor to a possible compensation for it. This second possible explanation is based to the fact that empathy deficits in autism are a function of interoceptive deficits related to alexithymia (Silani et al., 2008) and that alexithymia in turn has been found to be correlated with the confidence in emotion perception in Point-Light Displays (Lorey et al., 2012). In fact, Lorey et al. (2012) examined how the ability to perceive own emotions assessed with the Toronto Alexithymia Scale, is related to both the ability to perceive emotions depicted in PLDs and the confidence in these perceptions. The results showed that people with higher alexithymia scores were significantly less confident about their decisions, but did not differ from people with lower alexithymia scores in the valence of their ratings. Recent fMRI studies (e.g., Silani et al., 2008; Bird et al., 2010) have shown that the particular difficulties in emotional awareness in individuals with HAT are not related to their impairments in self-reflection/mentalizing but instead they are a function of interoceptive deficits related to alexithymia. Bird et al. (2010) suggest that the empathy deficits observed in autism may be due to the large comorbidity between alexithymic traits and autism, rather than representing a necessary feature of the social impairments in autism. Thus, if our HAT participants presented a high interoceptive deficit (as it is likely to be the case), this would explain why they did not rely on kinematic cues, being less confident than LAT ones in their judgment on PLDs (Lorey et al., 2012). This speculation is in need of further research, but it should be noticed that it does not exclude the other suggested possibility of a lack of confidence in judgements based on bodily kinematics. In both cases, in fact, it is possible that HAT individuals simply rely more on static cues because, if any thing, they may have been trained with emotional faces and not with emotional bodies.

A last possibility to explain why individuals with HAT do not use body cues to recognize fear like LAT ones, is based on the possible impairment in global motion which, as already suggested in this section, even if present, it does not emerge in this study (because of a long stimulus duration for PLDs) and could explain why HAT participants do not rely on bodily kinematics to recognize fear.

We think that a possible way to study biological motion perception in ASC without having to deal with long durations and motion coherence – which of course is involved in PLDs, not to mention the fact that PLDs with durations shorter than 1 s are difficult to see as emotional – would be to study the biological motion of a single point of light. Our suggestion is based on the idea that our capability to recognize biological motion is not strictly related to the dynamic template of the classical PLD, but rather to the kinematic structure of the movement of *each* single point (Runeson and Frykholm, 1981). In particular, our perceptual system is very well attuned to a peculiarity of human movement, namely, a particular relation between velocity and curvature known as the two-thirds power law (Lacquaniti et al., 1983). The sensitivity to this biological motion of a single point-of-light has been investigated in adults (e.g., Viviani and Stucchi, 1989; de’Sperati and Viviani, 1997; Actis-Grosso et al., 2001; Carlini et al., 2012) as well as in 4-day-old human neonates (using a standard preferential-looking paradigm, Méary et al., 2007) and indicates that human motion perception is attuned to biological kinematics. However, nobody has studied yet the biological motion of a single point-of-light in ASC. For example, findings from a preferential looking paradigm in 2-year-old toddlers indicate that only TD-children demonstrated a clear looking preference for biological PLDs, whereas toddlers diagnosed with autism did not (Klin et al., 2009). We think that a similar study with biological motion of a single point of light could rule out any possible involvement of motion coherence and duration, thus helping to solve the problem of different authors reporting different results in biological motion perception for the ASC group. It should also be noticed that a single point of light could also convey emotions, and could be studied accordingly with both TD and ASC populations. In fact, not only has it been shown that arm movements alone, performing simple actions, convey information about affect (Pollick et al., 2001), but it was recently found that specific motion patterns increase perceived intensity and arousal related to emotional faces (Chafi et al., 2012). Following this line of research, and taking into account recent evidence of a link between single dot kinematics and localizations (Actis-Grosso et al., 2008), we think that it would be possible to find specific kinematics (i.e., absolute velocity, accelerations, stops, and so on) related to specific emotions, so that a single point of light could be perceived as happier or sadder, in analogy with classical studies on animacy (Heider and Simmel, 1944; Michotte, 1954), helping in this way to better clarify the link between the perception of emotion (and, more in general, of agency as highlighted by studies on the so-called social network, Wheatley et al., 2007) and the perception of motion (Tavares et al., 2011). As a matter of fact, we think that future research should consider a new experiment focused on the perceived animacy and/or emotions of a single point, in order to study kinematic features of biological motion through short-duration stimuli.

In our view, the results in which HAT participants exhibited a different recognition pattern for fear, and were generally more inclined to use strategies based on processing static face details, could also account for the emotion recognition difficulty with static emotional faces often found in autistic population, in which recognition of fear is also found to be worse than in TD individuals (Uljarevic and Hamilton, 2013). What we suggest is that the recognition of emotions is based on kinematics even when static faces have to be judged. In fact, it has recently been suggested (Actis-Grosso and Zavagno, 2015) that pictures of emotional faces may convey information with respect to implied motion: namely the fact that a still photograph of an object in motion may convey dynamic information about the position of the object immediately before and after the photograph was taken (Freyd, 1983; Kourtzi and Kanwisher, 2000). Focusing on the facial expression of emotions, Actis-Grosso and Zavagno (2015) hypothesized that all emotions could be classified in terms of inherent dynamism, that might be a visible trace within the facial expression of an emotion (Freedberg and Gallese, 2007), and that some facial emotions are more visually dynamic than others. They asked a group of participants to rate both the emotional content and the dynamicity of emotional faces taken from static artworks and found that some facial emotions (i.e., disgust, anger, and fear) were positively related to the dynamicity attributed to the artworks, thus presenting a first evidence that also static emotional faces could be somehow dynamic, allowing the observer to extract dynamic information from their static representations. If this result is generalized across more different emotions and with photographs such as the ones used in this study, we think that it would be possible to find a specific impairment for ASC in recognizing “dynamic” emotions in static pictures.

## Conclusion

This study highlights for the first time that certain emotions are expressed and perceived better through dynamic information whereas others are better recognized through static ones and that LAT individuals and individuals with HAT based their emotion recognition on different cues. We thus think that future research rather than searching for a universal and primary emotion recognition impairment in autism should take into account that different emotions are better recognized though different stimulus types which are processed differently, in LAT individuals and individuals with HAT. We also think that the present study, besides sheding some light on the link between the perception of motion and the perception of emotion in HAT individuals, suggests some future directions for both scientific research – that should study in more detail the kinematics associated with single emotions and the way in which individuals with ASC rely on it to recognize emotions – and clinical training – that should be more focused on body movement.

## Conflict of Interest Statement

The authors declare that the research was conducted in the absence of any commercial or financial relationships that could be construed as a potential conflict of interest.

## Footnotes

1 The choice to compare only four basic emotions out of the six typically considered as basic (see Ekman and Friesen, 1971) is due to the fact that both disgust and surprise have been found (e.g., Dittrich et al., 1996; Atkinson et al., 2004) to be easily confounded with other emotions (surprise also being considered a mixed emotion) and for this reason we preferred not to include them in our study.
